# Adaptive MCS selection and resource planning for energy-efficient communication in LTE-M based IoT sensing platform

**DOI:** 10.1371/journal.pone.0182527

**Published:** 2017-08-10

**Authors:** Nhu-Ngoc Dao, Minho Park, Joongheon Kim, Sungrae Cho

**Affiliations:** 1 School of Computer Science and Engineering, Chung-Ang University, Seoul, South Korea; 2 Department of Information Communication, Materials, and Chemistry Convergence Technology, Soongsil University, Seoul, South Korea; Northwestern Polytechnical University, CHINA

## Abstract

As an important part of IoTization trends, wireless sensing technologies have been involved in many fields of human life. In cellular network evolution, the long term evolution advanced (LTE-A) networks including machine-type communication (MTC) features (named LTE-M) provide a promising infrastructure for a proliferation of Internet of things (IoT) sensing platform. However, LTE-M may not be optimally exploited for directly supporting such low-data-rate devices in terms of energy efficiency since it depends on core technologies of LTE that are originally designed for high-data-rate services. Focusing on this circumstance, we propose a novel adaptive modulation and coding selection (AMCS) algorithm to address the energy consumption problem in the LTE-M based IoT-sensing platform. The proposed algorithm determines the optimal pair of MCS and the number of primary resource blocks (#PRBs), at which the transport block size is sufficient to packetize the sensing data within the minimum transmit power. In addition, a quantity-oriented resource planning (QORP) technique that utilizes these optimal MCS levels as main criteria for spectrum allocation has been proposed for better adapting to the sensing node requirements. The simulation results reveal that the proposed approach significantly reduces the energy consumption of IoT sensing nodes and #PRBs up to 23.09% and 25.98%, respectively.

## 1 Introduction

**Fig 1:**
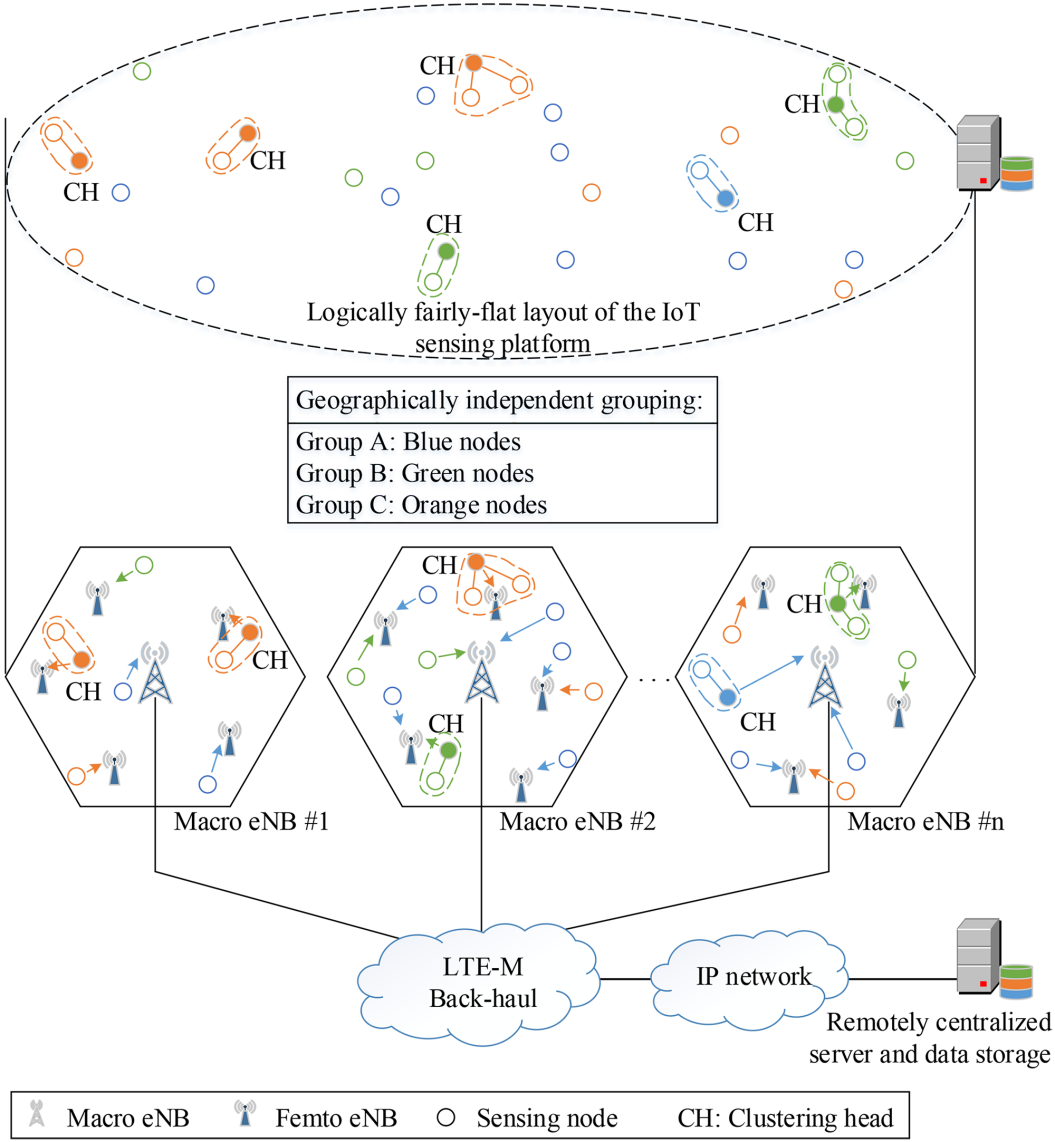
Architecture of the LTE-M-based IoT-sensing platform.

Cellular standardization organizations such as 3GPP (the third generation partnership project) are actively working towards the next stages of the long term evolution (LTE) standard in order to support machine-type communication (MTC), also known as LTE-M. MTC defines data communication among devices without human aids, which mainly applies to low-data-rate and low-power devices or things. According to this, MTC is considered as a promising approach for the spreading of Internet of things (IoT) sensing platforms that generally involve small data communication [1]. Utilizing the benefits of the LTE-M infrastructure, IoT sensing platforms (IoTSPs) are expected to cover a larger geographical area with an economically feasible cost since MTC has a close relationship with IoT devices [2]. In the scope of this paper, the term IoT sensing platform is equivalent to the term IoT sensing system. As a result, the LTE-M based IoTSPs provide (i) remotely centralized data processing and storage, (ii) geographically independent grouping, and (iii) a logically fairly-flat network layout where LTE-M sensing nodes are able to be connected using a wireless connection via eNBs. Fig 1 illustrates the typical architecture of an LTE-M based IoT-sensing platform. In the lower layer, sensing nodes and clustering heads portably associate with the LTE-M eNodeBs via a wireless interface. Utilizing the LTE-M infrastructure, the sensing nodes communicate with each other and connect to both the remotely centralized servers and the data storage in order to exploit the sensing data. It is worth noting that the communications between the sensing nodes and between the sensing node and the servers/storage are indirectedly established without location concerns. As abstracted in the upper layer, all sensing nodes are connected within a logically fairly-flat layout, including the remotely centralized servers and data storage.

In addition to the many advantages of utilizing an LTE-M infrastructure for IoTSPs, the challenges are also considerable, especially in terms of energy efficiency. Although a large variety of energy-efficient techniques have been proposed in cellular networks, there are only a limited number of schemes for LTE-M. These existing LTE-M based energy-efficient techniques can be classified into the following categories: (i) power control [3, 4], (ii) scheduling [5–9], and (iii) data reduction [10–15]. Directly tackling energy consumption, power control methods [3, 4] focus on adapting the transmit power levels to time-varying channel conditions. These adaptations can be performed using a channel measurement report procedure. In comparison, the scheduling approaches [5–9] reschedule time-based operations that are negotiated between the devices and the network regarding sleep/wakeup modes, tracking periods for device position report, and channel measurement periods for environmental monitoring. These operations are dynamically optimized based on the mobility features of the devices. The data reduction approach [10–15] aims to save energy by reducing the amount of unnecessary data and overhead. Data aggregation/compression (i.e., data carrying ratio) and effective coding/routing (i.e., overhead reduction) are prime examples of data reduction. A detailed survey of the related work is provided in Section 2. According to the taxonomy, our proposed technique falls under data reduction approaches. With respect to the MCS level, our scheme considers transmission data size as an important factor for adapting to uplink transmission to reduce the transmit power.

Although 3GPP has already introduced some new enhanced power modes and more efficient signaling techniques to better support MTC directly through the LTE infrastructure [16, 17], the transmission data size has not been considered in the modulation and coding scheme (MCS) negotiation process, which might lead to power wastage. In general, MCS implements the channel quality (e.g., interference level, receive power, and signal-to-interference-plus-noise ratio). In other words, when the channel quality is good, a higher MCS level is assigned, or vice versa. For a higher MCS level, a larger transport block (TB) is transmitted in LTE-M [18]. However, the TB might be over-sized for the transmission of the small sensory information typical in IoTSPs. This problem causes significant energy consumption on LTE-M devices (which will be discussed in more detail in Section 3). Therefore, a transmission data size adaptation for the MCS negotiation process is crucial.

Through the analysis in Section 3, it is seen that the energy consumption can be minimized using an optimal pair of MCS and the number of primary resource blocks (#PRBs) for each given size of transmission data. First, we propose an adaptive MCS selection (AMCS) mechanism where the sensing node adaptively updates the channel quality indicator (CQI) and power headroom report (PHR) to the femto eNB (FeNB) with respect to the size of the sensing data in order to achieve an optimal pair of MCS and #PRBs. The optimal MCS and #PRBs are determined by the minimum MCS level and #PRBs, at which the transport block size is sufficient to packetize the sensing data within the minimum transmit power. This results in an optimal MCS that is always lower quality or equal to the typical LTE-based MCS. If less optimal MCS is achieved, the sensing node can operate at lower channel conditions. Therefore, we additionally propose a quantity-oriented resource planning (QORP) technique that utilizes these optimal MCS levels of sensing nodes as the criteria to re-plan available channels among the neighboring FeNBs instead of directly considering the channel interference among them. Since the optimal MCS correspondingly represents the requirements of the sensing node for successful data transmission, the proposed resource planning scheme better adapts to the sensing node requirements. Intensive analysis and evaluation show that the channel reuse ratio significantly increases among the FeNBs, resulting in a larger #PRBs.

Our contributions in this paper are described as follows:

- The adaptive MCS selection (AMCS) algorithm renegotiates the optimal values of MCS level and #PRBs for the sensing node to reduce energy consumption in the uplink channel without negative impacts on the transmission requirements.
- Based on a focused consideration on these optimal MCS levels, the quantity-oriented resource planning (QORP) algorithm better adapts to the sensing node requirements and achieves a higher channel reuse ratio among FeNBs.
- Moreover, the interference caused by the sensing nodes is also mitigated because the uplink transmit power is reduced due to the optimal MCS. As a result, the quality of the uplink channel is improved and the throughput is proportionally increased.

The remainder of this paper is organized as follows. We survey the existing related work in Section 2. Section 3 discusses the energy-efficiency problem for small data communication in LTE-M-based IoTSPs. In Section 4, we describe the proposed algorithms along with representative toy models. We also provide the performance evaluation and discussion in Section 5. Finally, we draw conclusions in Section 6.

## 2 Related work

First, we briefly summarize the existing techniques standardized by 3GPP for MTC devices. Since Release 12, 3GPP has standardized an enhanced power save mode (PSM) that defines a suitable procedure allowing the connected devices to remain registered with the network in order to reduce the signal required for modem wakeup. In the PSM, the devices can turn off their transceivers to avoid power consumption during idle time [19, 20]. The maximum sleep cycle accepted in LTE networks is 2.56 seconds, which is very short in comparison to most MTC schemes. To address this inefficiency, extended discontinuous reception (DRX) [21] has been introduced to extend the sleep cycles by up to several minutes in order to eliminate unnecessary signaling as well as provide an extended battery life to the devices. On the other hand, less frequent tracking area updates and measurements are utilized to reduce the location updates and channel measurement messages on the device side. In addition to the variety of common resource management techniques used in LTE-A networks for energy efficiency (e.g., inter-cell interference mitigation, PRB schedulers, or frequency planning) [22], 3GPP has defined a connectionless random access channel (RACH) [23], which enables the connected devices to transmit data via a common channel for more efficient transition between the data transferring and signaling states.

Based on the taxonomy mentioned in Section 1, a hybrid scheduler proposed by Lauridsen *et al.* [3] falls under the power control approach, which adjusts the device transmit power by scheduling the uplink PRBs in both the frequency and time domains in order to determine whether a low transmit power within a long transmission time or a high transmit power within a short transmission time would be the most energy-efficient strategy. In [4], Yang *et al.* proposed a distributed power control that modifies the device transmit powers to satisfy a Bayesian Nash equilibrium where the power consumption is minimal in the network. The equilibrium is determined by utilizing non-cooperative game theoretic analysis.

Based on the 3GPP DRX scheduler, Balasubramanya *et al.* proposed DRX with quick sleeping [5], where a quick sleeping indication (QSI) indicates whether the MTC devices can sleep early, which can only occur when there is no valid incoming page from the network. Using the coverage enhancement (CE) feature, the QSI included in the synchronization signal is transferred across the physical broadcast channel when the CE is supported. Otherwise, a number of dedicated resources on the physical downlink shared channel are assigned for QSI delivery. In [6], an energy-efficient sleep schedule proposed by Liang *et al.* balances the impacts between the quality of service (QoS) parameters and the DRX configurations in order to maximize the MTC devices’ sleep periods so as to save energy while satisfying their QoS requirements in terms of traffic bit-rate, packet delay, and packet loss rate. On the other hand, Jin *et al.* proposed an active DRX mechanism [7] that influences the control of the downlink transmission, where the system would go to sleep only when there is no data frame arrival within a predefined sleep-delay timer. In [8], Wang *et al.* introduced a switching rule based on the rationale that a long DRX cycle is well suited for power-efficient operation during periods of low device activity, and vice versa. Therefore, a short DRX cycle is appropriate for low latency and devices that actively transfer data. In [9], Zhou *et al.* proposed effective resource allocation in cloud-based radio access network among remote radio head in order to achieve energy efficiency by using noncooperative game theoretic model.

By utilizing data reduction for energy efficiency purposes, Kim *et al.* [10] improved the performance of the uplink power control by adapting the power headroom report with respect to the device power capability to achieve a proper #PRBs from the network. Since #PRBs are efficiently allocated, the transmission energy per PRB ratio is significantly increased, resulting in a lower transmission overhead. In [11], Andreev *et al.* proposed a contention-based LTE transmission (COBALT) mechanism that allows the devices to transmit small data packets directly over the physical uplink shared channel (PUSCH) instead of spending extra signaling overhead on LTE common dedicated control channels (i.e., physical uplink control channel and physical random access channel). Aghili *et al.* [12] suggested a wireless transmit/receive unit (WTRU) that autonomously releases the connection of small data appended to a control plane message without any extra control signals from the network. Using the user social pattern model, Zhang *et al.* [13] characterized the general user behaviors, patterns, and rules of user groups in a social manner as optimization parameters for enhancing spectral-energy efficiency. In [14], Huang *et al.* considered the connection topology to optimize energy consumption in large-scale with focusing on IoT devices. On the other hand, Arouk *et al.* proposed the further improvement-traffic scattering for group paging (FI-TSFGP) which aims to improve the performance of group paging when the number of MTC device is high. This grouping technique significantly reduces both channel access latency and energy consumption [15].

Although 3GPP Rel. 12 and 13 have already standardized numerous techniques in order to satisfy IoT devices, the transmission data size has not been considered in the modulation and coding scheme (MCS) negotiation process. By considering the transmission data size, our proposed scheme better adapts to the IoT sensing node requirements. As a result, the transmission energy and the number of assigned PRBs for IoT sensing nodes are both significantly decreased. It is worth noting that the proposed algorithms can be implemented with most existing techniques regardless of the MCS-PHR negotiation processes.

## 3 Problem statement

In the LTE-M network, devices transmit data over the physical uplink shared channel (PUSCH) using an uplink power control (UPC) with transmit power *P*_tx_ following the 3GPP standard [18] as:

$$\begin{array}{c} P_{\text{tx}}=\min\{\begin{array}{c} P_{\max},\;\;\;\;\;\;\;\\ 10\log_{10}\left(N\right)+P_{0}+\alpha L+\Delta_{MCS}+\gamma \end{array}\}, \end{array}$$ (1)

where *P*_max_ is the allowable maximum transmit power of the device, *N* represents an instantaneous bandwidth measured by #PRBs allocated to the device, *P*_0_ defines the desired received power at an FeNB to properly demodulate and decode the received signal within a given reliability, *L* is the downlink path loss estimated by the device, and *α* is a path loss compensation factor (0 ≤ *α* ≤ 1). The portion *P*_0_ + *αL* forms an open-loop power control. The remainder is related to the closed-loop power control including Δ_*MCS*_ and *γ*, where Δ_*MCS*_ is an MCS-dependent power offset that represents the difference in power between the target MCS and basic MCS. This means that if the device uses a higher target MCS than the basic one, the Δ_*MCS*_ value is positive, and the corresponding transmit power should increase, or vice versa. Lastly, *γ* describes the transmit power control (TPC) command issued by the FeNB in order to adjust the uplink transmit power.

MCS is used to adapt to the time-varying uplink channel condition. In 3GPP Rel. 12, three modulation schemes are exploited, i.e., quadrature phase shift keying (QPSK), 16 quadrature amplitude modulation (QAM), and 64 QAM. If the quality of the channel is very good, 64 QAM can be used in order to modulate 6 bits of information into 1 symbol to transmit. Otherwise, 16 QAM or QPSK will be used to execute 4 bits or 2 bits of information per symbol. Furthermore, channel coding schemes are also selected based on the channel conditions in order to guarantee proper demodulation and decoding performance of the received signal at the FeNB. The FeNB mainly decides the target MCS value using the CQI that is reported from the device. The details on mapping tables *f*(⋅) and *g*(⋅) from the signal-to-interference-plus-noise-ratio (SINR) to CQI and from CQI to MCS, respectively, were introduced in [24, 25]. We generalized them as follows:

$$\begin{array}{c} CQI=f(SINR), \end{array}$$ (2)

$$\begin{array}{c} MCS=g(CQI). \end{array}$$ (3)

If the uplink resources negotiated between FeNB and the device are not enough to transmit the required amount of data, the device calculates its power headroom (PH) and reports the result to the FeNB to request a higher #PRBs [21]. *PH* is derived from *P*_max_ by subtracting the calculated transmit power as follows:

$$\begin{array}{c} PH=P_{\max}-\left(10\log_{10}\left(N\right)+P_{0}+\alpha L+\Delta_{MCS}+\gamma \right). \end{array}$$ (4)

**Fig 2:**
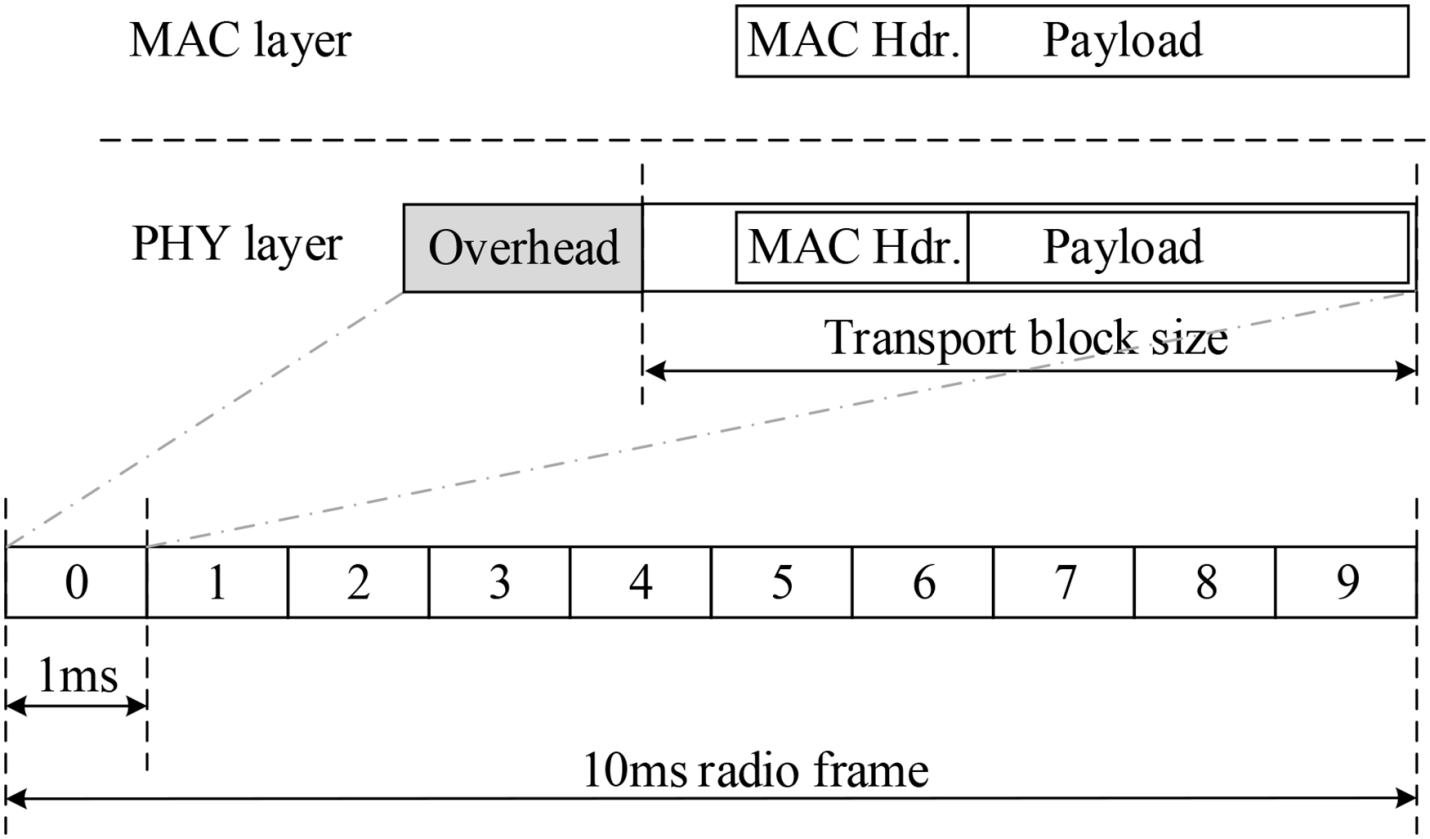
Relationship between the required transmission data and the transport block size.

In this paper, we consider the total MAC layer data as the required transmission data. Fig 2 illustrates the data transmission from the medium access control (MAC) layer to the physical (PHY) layer. The required transmission data is modulated and encoded into a physical TB during each 1 ms. The transport block size (TBS) depends on the selected MCS and #PRBs [6].

The aforementioned resource allocation mechanism is suitable for normal LTE devices that transfer a huge volume of data at high speeds. However, the default mechanism may not be optimal for sensing nodes that require small data communications and low power operations. For instance, the application-layer data of a smoke detector has only one bit of binary information indicating whether smoke is detected. This information is packetized with an additional header and trailer from the application layer to the physical layer, resulting in a 280-bit MAC frame [26]. Assuming the highest available MCS—level of 28 and 1 PRB (i.e., TBS equal to 712 bits, see Table 1) are assigned, the 280-bit MAC frame occupies just 39.33% of the assigned 712-bit TB, causing 60.67% overhead excluding the header and channel coding redundancies. Table 1 shows that utilization of the lower transmit power resulting from a level 16 MCS and 1 PRB (i.e., TBS equal to 280 bits) is the best choice for this data transmission, where the MAC frame occupies 100% of the assigned TB. Since a lower MCS is used in this case, the sensing node consumes less power (see Eq 1).

**Table 1:** A sample of the transport block size in bits defined in 3GPP TS 36.213 [18].

MCS	TBS index	#PRB						
		1	2	3	4	5	6	…
…	…	…	…	…	…	…	…	…
7	7	104	224	328	472	584	712	…
8	8	120	256	392	536	680	808	…
9	9	136	296	456	616	776	936	…
10	9	136	296	456	616	776	936	…
11	10	144	328	504	680	872	1032	…
12	11	176	376	584	776	1000	1192	…
13	12	208	440	680	904	1128	1352	…
14	13	224	488	744	1000	1256	1544	…
15	14	256	552	840	1128	1416	1736	…
16	15	280	600	904	1224	1544	1800	…
17	15	280	600	904	1224	1544	1800	…
18	16	328	632	968	1288	1608	1928	…
19	17	336	696	1064	1416	1800	2152	…
20	18	376	776	1160	1544	1992	2344	…
21	19	408	840	1288	1736	2152	2600	…
22	20	440	904	1384	1864	2344	2792	…
23	21	488	1000	1480	1992	2472	2984	…
…	…	…	…	…	…	…	…	…
27	25	616	1256	1864	2536	3112	3752	…
28	26	712	1480	2216	2984	3752	4392	…

**Fig 3:**
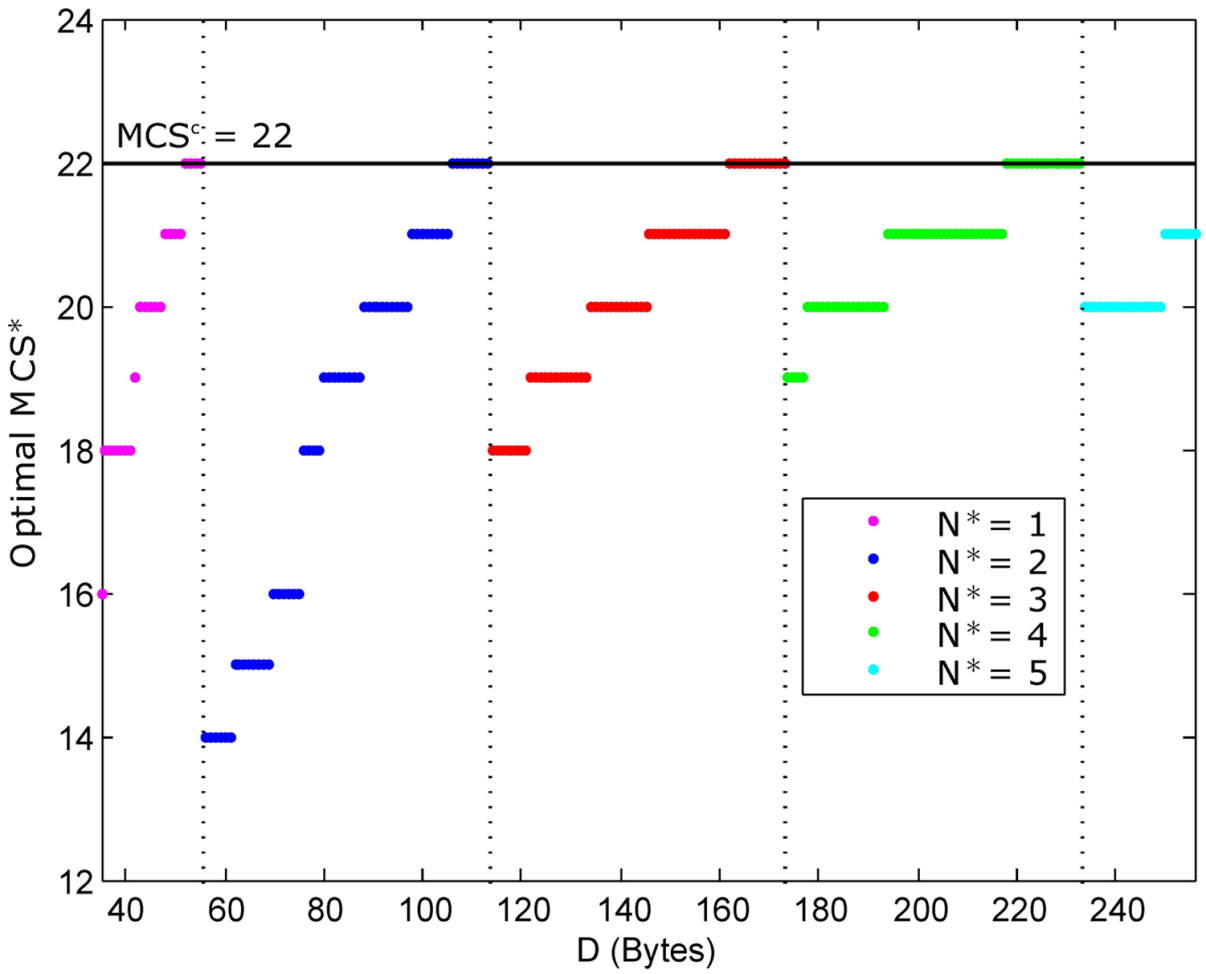
The achievable optimal pair (MCS*, N*) constrained by a *MCS*^*c*^ of 22.

Without loss of generality in terms of the energy efficiency, the optimal MCS and #PRBs (denoted by MCS* and N*, respectively) for sensing nodes can be expressed as follows:

$$\begin{array}{c} \left(MCS^{*},N^{*}\right)=\underset{\begin{array}{c} 0\;\leq \;MCS\;\leq \;26\\ 1\;\leq \;N\;\leq \;110 \end{array}}{\arg\min}\left(10\log_{10}\left(N\right)+\Delta_{MCS}\right), \end{array}$$ (5)

subject to

$$\begin{array}{c} \left\{ \begin{array}{c} \text{TBS}(MCS^{*},N^{*})\geq D\\ MCS^{*}\leq MCS^{c} \end{array},\right. \end{array}$$ (6)

where *D* and *MCS*^*c*^ denote the transmission data in bits and the current MCS level, respectively. For instance, assuming that *D* and *MCS*^*c*^ are 100 Bytes (i.e., 800 bits) and 22, respectively, the TBS candidates should be greater than or equal to 800, as highlighted in Table 1. Due to the constraint MCS* ≤ *MCS*^*c*^, the red marked numbers are ignored, resulting in only the applicable yellow and cyan marked ones. Among the remaining color marked numbers, the optimal pair (MCS*, N*) is derived from the one that consumes the minimum transmit power of 10log_10_(*N*) + Δ_*MCS*_, i.e., (MCS*, N*) = (21, 2), corresponding to the 840-bit TBS (cyan marked one). As a result, Fig 3 represents the optimal (MCS*, N*) depending on the variable data size *D* with a current MCS level of 22. It is observed that the number of PRBs (N*) increases proportionally with the data size *D* while the optimal MCS* varies based on *D* and N*.

Generally, the power consumption *E* used to transfer data is given by:

$$\begin{array}{c} E=P_{\text{tx}}\times T, \end{array}$$ (7)

where *T* is the time duration in seconds to transfer data and *P*_tx_ is the transmit power (Eq 1). From Eqs 1 and 5, the energy *E* is derived as:

$$\begin{array}{c} E=10^{\frac{P_{\text{tx}}\left(MCS^{*},\;N^{*}\right)}{10}-3}\times T. \end{array}$$ (8)

Hence, the energy efficiency (*χ*) that represents how much energy *E* is consumed to successfully transmit a given data size *D* is given by:

$$\begin{array}{c} \chi =\frac{D}{E}. \end{array}$$ (9)

In the next section, we propose an appropriate solution to obtain the optimal (MCS*, N*) and utilize it for a better energy efficiency *χ* in LTE-M based IoTSPs.

## 4 The proposed algorithms

### 4.1 Adaptive MCS-PHR selection algorithm

In Section 3, we discussed how the MCS level that the FeNB assigns to a sensing node based on CQI is not always optimal for small data communications. Therefore, we proposed an optimal pair of MCS level and #PRBs (MCS*, N*) to minimize the energy consumption for data transmission. In this section, an adaptive MCS selection algorithm (AMCS) is introduced to utilize available free resources of the FeNB to satisfy the optimal pair (MCS*, N*) (see Algorithm 1).

When a sensing node has data to transmit, the node calculates its own optimal value (MCS*, N*). If the MCS* is equal to the current *MCS*^*c*^ (i.e., the current *MCS*^*c*^ is optimal), the device does not need to change this value. Subsequently, the sensing node requires N* PRBs to successfully transmit the data *D*. If the current number of PRBs *N*^*c*^ is equal to N* (i.e., #PRBs is large enough), the sensing node modulates and encodes data *D* into the TBS. Otherwise, the sensing node has to calculate and report its power headroom *PH* to the FeNB in order to request more #PRBs.

**Algorithm 1**: **Adaptive MCS-PHR selection**.

1 *D* ← the required transmission data;

2 *MCS^c^*, *N^c^* ← the current MCS and #PRBs;

3 MCS*, N* ← the optimal MCS and #PRBs;

4 **foreach** *MCS renegotiation* **do**

5  **if** *has D bits data to transmit* **then**

6   Find (MCS*, N*) by Eq 5;

7   **if** MCS* = *MCS^c^* **then**         /*Current MCS is optimal*/

8    **if** N* = *N*^*c*^ **then**          /* Current #PRBs are enough */

9     Modulate and encode the data *D* into the TBS;

10    **else**             /* Current #PRBs are not enough */

11     Calculate *PH* by Eq 4;

12     Send *PHR* to the FeNB;

13     Modulate and encode the data *D* into the TBS;

14    **end**

15   **else**              /* Current MCS is not optimal */

16    Send *CQI* update to the FeNB;

17    Calculate *PH* by Eq 4;

18    Send *PHR* to the FeNB;

19    Receive *N*^*a*^ from the FeNB;

20    Modulate and encode the data *D* into the TBS;

21    **if** *N*^*a*^ < N* **then**       /* The FeNB has insufficient #PRBs */

22     Return to LTE-M based MCS selection;

23     Set the timer to *τ* ms;      /* Stop the algorithm in *τ* ms */

24    **end**

25   **end**

26  **end**

27 **end**

If the current *MCS*^*c*^ does not equal MCS*, then the sensing node sends a CQI-update message to the FeNB to re-negotiate the optimal MCS*. After obtaining an appropriate MCS*, the sensing node continues to send PHR report messages to ask the FeNB to request a #PRBs of N*. Based on the PHR report, the FeNB will allocate the required #PRBs to the sensing node if free resources are available.

However, if the available resources are not sufficient to be assigned to the sensing node as required (denote *N*^*a*^ as the assigned #PRBs), the sensing node should return to the general LTE-M based MCS selection procedure for a successful data transmission in the next 1 ms transmission time interval (TTI). The proposed AMCS algorithm is paused in *τ* ms to transfer all of the buffered data within the general LTE-M-based MCS.

### 4.2 Quantity-oriented resource planning algorithm

According to the AMCS algorithm, the interference caused by the sensing nodes is also mitigated because the uplink transmit power is reduced as a result of obtaining the optimal MCS. From the perspective of resource-planning decision making, if a sub-channel is able to support a given MCS level for the sensing nodes (i.e., reflected by the SINR), we can assume that the sub-channel is clear enough, and thus the interference on the sub-channel is not considerable. Therefore, this sub-channel can be simultaneously assigned to neighboring FeNBs. Taking advantage of the optimal MCS levels, we proposed a quantity-oriented resource planning (QORP) algorithm that adapts better to the sensing node requirements and achieves a higher channel reuse ratio among the FeNBs. Let us define the given MCS level as an MCS threshold denoted by *MCS*_*thres*_ as follows:

$$\begin{array}{c} MCS_{thres}=\lceil \frac{1}{N_{s}}\sum_{i=1}^{N_{s}}MCS_{i}^{*}+\delta \rceil , \end{array}$$ (10)

where *N*_*s*_ is the total number of sensing nodes serviced by the FeNBs and *δ* is a correction factor.

In this paper, we assume that the 5G HetNets utilize typical optimal fractional frequency reuse (OFFR) for MeNB resource planning [27]. The target of OFFR is to optimize the total capacity of the network. Among the FeNBs, the relationship between each pair of two FeNBs is assumed to have considerable interference if the average MCS of the sensing nodes in the coverage of the two FeNBs is less than the threshold *MCS*_*thres*_. The corresponding condition is described as follows:

$$\begin{array}{c} \frac{1}{N_{s}}\sum_{i=1}^{N_{s}}MCS_{i}\leq MCS_{thres}. \end{array}$$ (11)

To plan resource allocation for the FeNBs, we first transform the FeNB positions into a graph model where each FeNB is a vertex. Based on Eq 11, if the interference between two neighboring FeNBs is considerable, we use an undirected edge to connect the two corresponding vertices. For each sub-channel, the maximum independent set (MIS) method [28] is utilized to identify the maximum set of FeNBs where no FeNB interferes. The proposed quantity-oriented resource planning (QORP) method is described in Algorithm 2 as follows.

- (Step 1) The FeNB system is transformed into an undirected graph *G(V, E)*, where *V* is a set of FeNBs and *E* is a set of interference edges specified by Eq 11.
- (Step 2) For each sub-channel *s* ∈ *S* (*S* is the set of all sub-channels), we draw a corresponding graph *G*_*s*_(*V*_*s*_, *E*_*s*_) that contains all FeNBs that are possibly assigned with sub-channel *s*.
- (Step 3) The MIS method is applied to the graph *G*_*s*_(*V*_*s*_, *E*_*s*_) to identify the maximum set of independent FeNBs on sub-channel *s*.
- (Step 4) The algorithm is repeated from (Step 2) for all sub-channels in the set *S*. The final result is an adjacency matrix of optimal sub-channels *S* and FeNBs *V*.

**Fig 4:**
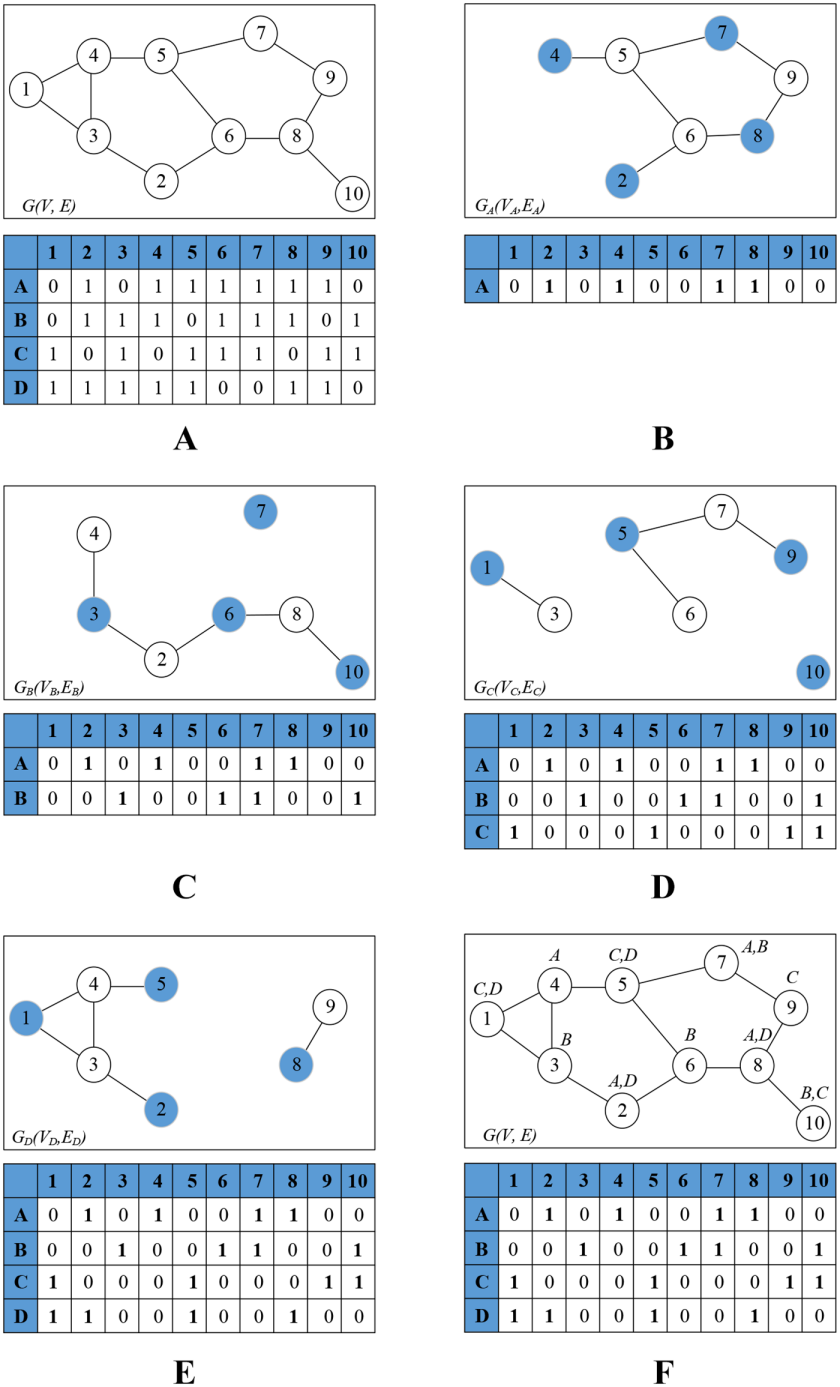
Example of the quantity-oriented resource planning algorithm. A) The graph *G(V, E)* of the FeNB system and the adjacency matrix Φ_*S*,*V*_ of possible sub-channels assignment for FeNBs. B) QORP algorithm for sub-channel *A* and the adjacency matrix Ψ_*S*,*V*_ of optimal sub-channel assignment. C) QORP algorithm for sub-channel *B* and the updated adjacency matrix Ψ_*S*,*V*_. D) QORP algorithm for sub-channel *C* and the updated adjacency matrix Ψ_*S*,*V*_. E) QORP algorithm for sub-channel *D* and the updated adjacency matrix Ψ_*S*,*V*_. F) The final adjacency matrix Ψ_*S*,*V*_ of the optimal sub-channel assignment for graph *G(V, E)*.

For example, consider a 10-FeNB system and a set of 4 possible sub-channels {*A*, *B*, *C*, *D*}, as shown in Fig 4A. The FeNB system is transformed into an undirected graph model where the vertices represent FeNBs and the edges represent the interference between two corresponding FeNBs. In the adjacency matrix *Φ*_*S*,*V*_ of possible sub-channel assignments for FeNBs, the columns represent 10 FeNBs, named 1 to 10, and the rows represent 4 sub-channels, named *A*, *B*, *C*, and *D*. The entries have a value of 1 if the corresponding FeNB (indexed by column name) is possibly assigned with the corresponding sub-channel (indexed by row name); otherwise, the entries have a value of 0.

In Fig 4B and considering sub-channel *A*, the proposed QORP algorithm shows that the set of independent vertices {2, 4, 7, 8} is the maximum independent set of the graph *G*_*A*_(*V*_*A*_,*E*_*A*_); therefore, sub-channel *A* should be simultaneously assigned to FeNBs 2, 4, 7, and 8 without interference.

**Algorithm 2**: **Quantity-oriented resource planning**.

**Input**: A set of FeNBs’ position *V*, a set of all sub-channels *S*, and an adjacency matrix Φ_*S*,*V*_ resulting from OFFR.

**Output**: An adjacency matrix Ψ_*S*, *V*_ represents the optimal assigned sub-channels to FeNBs.

1 Initialize a graph *G(V, E)*;

2 **foreach** *a pair of V*_*i*_ *and V*_*j*_; *i* ≠ *j*; 1 ≤ *i*, *j* ≤ *N*_*f*_ **do**   /* Calculate graph model *G*(*V*, *E*)) */

3  **if** *the* Eq 11 == *TRUE* **then**

4   *E*_*i*,*j*_ = 1;

5  **else**

6   *E*_*i*,*j*_ = 0;

7  **end**

8 **end**

9 Initialize Ψ_*S*,*V*_;

10 **foreach** *s* ∈ *S* **do**        /* Calculate the adjacency matrix Ψ_*S*,*V*_ */

11  **foreach** *v* ∈ *V* **do**              */ Calculate *G*_*s*_(*V*_*s*_, *E*_*s*_ */

12   **if** Φ[*s*][*v*] = = 1 **then**

13    *G*_*s*_(*v*, *E*_*s*_) = *G*(*v*, *E*);

14   **else**

15    *G*_*s*_(*v*, *E*_*s*_) = (0, 0);

16   **end**

17  **end**

18  Apply MIS algorithm for *G*_*s*_(*V*_*s*_, *E*_*s*_);

19  Append the output to Ψ_*S*,*V*_;

20 **end**

Performing the same processes for sub-channels *B*, *C*, and *D*, we obtain the corresponding results in Fig 4C, 4D and 4E, respectively. Fig 4F shows the final adjacency matrix *Ψ*_*S*,*V*_ of the optimal sub-channel assignment for the graph *G(V, E)*.

## 5 Performance evaluation

### 5.1 Simulation setup

We developed a simulation network including 5 well-planned MeNBs without considerable inter-cell interference. The number of FeNBs per MeNB is randomly deployed in the range of [20, 300] to determine the influence of FeNB density on the effectiveness of the proposed algorithm. Table 2 lists the detailed parameters used to evaluate the network performance. The parameter values comply with the 3GPP TS25.104 and TR36.814 standards [30, 31]. The channel gains for uplink and downlink are included in the transmit power and receiver sensitivity, respectively. The numerical input dataset is provided in the Supporting information section, S1 Dataset.

**Table 2:** Simulation parameters.

Parameters	MeNB	FeNB
Number of cells	5	20–300 per MeNB (*N*_*f*_)
Transmit power	43 dBm on the primary channels 46 dBm on the secondary channels	15 dBm
Receiver sensitivity	-121 dBm	-107 dBm
Path loss model	3GPP outdoor: *L* = 15.3 + 37.6*log*_10_(*R*) (dB)	3GPP indoor: *L* = 38.46 + 20*log*_10_(*R*) + *L*_*wall*_ (dB)
Bandwidth	20 MHz	
Frequency	2000 MHz	
*P*_*max*_	23 dBm	
*α*	0.9	
*γ*	0	
sensing node’s receiver sensitivity	-100 dBm [29]	
Data packet size (*D*)	35–256 Bytes	

The simulation processes include contiguous steps as follows:

- **Step 1**: To analyze numerous scenarios, we deployed various number of FeNBs in amounts of {20, 40, 60, …, 300} corresponding to each scenario since the FeNB density significantly affects the wireless channel quality, which drives the MCS negotiation processes.
- **Step 2**: Within each networking scenario provided in Step 1, the data packet size (*D*) issued by the sensing nodes was adjusted following the packet sizes of {35, 55, 75, …, 215, 235, 256}. Along with the common simulation parameters shown in Table 2, selective numbers of FeNBs and data packet sizes form a variety of networking case studies.
- **Step 3**: The proposed QORP algorithm is compared with (i) the OFFR scheme [27] and (ii) the graph coloring-based cognitive spectrum allocation (GC-CSA) scheme [32] in terms of the energy efficiency of the sensing nodes and the average number of resource blocks assigned to each sensing node. The results are gathered based on all case studies.

### 5.2 Simulation results

**Fig 5:**
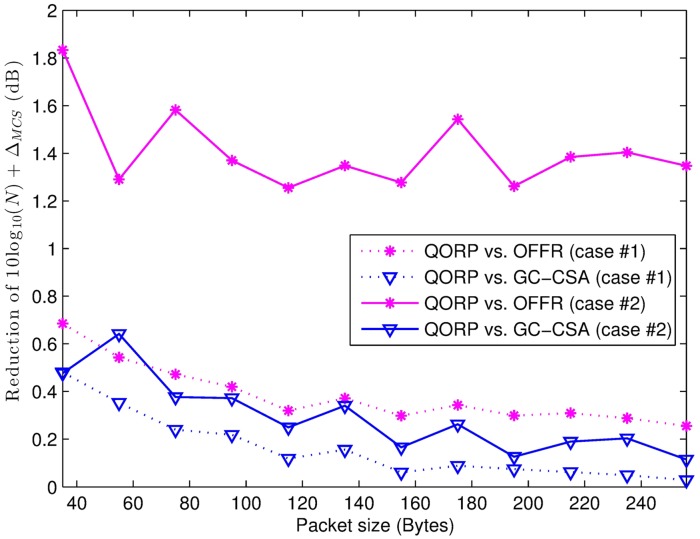
The reduction of 10log_10_(*N*) + Δ_*MCS*_ in the uplink transmit power. The number of FeNBs in cases #1 and #2 is 50 and 300, respectively.

Fig 5 represents the reduction part 10log_10_(*N*) + Δ_*MCS*_ in the uplink transmit power according to packet size. The trend of the line graphs shows that our proposed algorithm obtains a better performance when the packet size is small. For small packet sizes, the minimum MCS index that is required to successfully transmit the packet is also small, and the Δ*MCS* is higher; this is because of the reduction from the assigned MCS index to the required MCS index (i.e., the optimal value MCS*).

In case #1, the number of FeNBs is 50 per macro cell, which creates low inter-cell interference but also lowers the receiver power due to the long distance from the sensing nodes to their associated FeNBs. This leads to a worse SINR and low MCS, resulting in a low Δ*MCS*. On the other hand, in case #2, the FeNB density is high (the number of FeNBs is 300 per macro cell), causing a higher inter-cell interference. Because the GC-GSA algorithm and our proposed algorithm mitigate most interference, they provide better SINR and MCS, however our proposed algorithm achieves a better Δ*MCS* in terms of the energy efficiency. The statistical indexes are summarized in Table 3.

**Table 3:** The statistical index of the reduction part 10log_10_(*N*) + Δ_*MCS*_ in the uplink transmit power.

Index	QORP vs. OFFR (50 FeNBs)	QORP vs. GC-CSA (50 FeNBs)	QORP vs. OFFR (300 FeNBs)	QORP vs. GC-CSA (300 FeNBs)
Maximum reduction (dB)	0.685	0.481	1.833	0.642
Minimum reduction (dB)	0.255	0.028	1.255	0.115
Average (dB)	0.384	0.161	1.408	0.293

**Fig 6:**
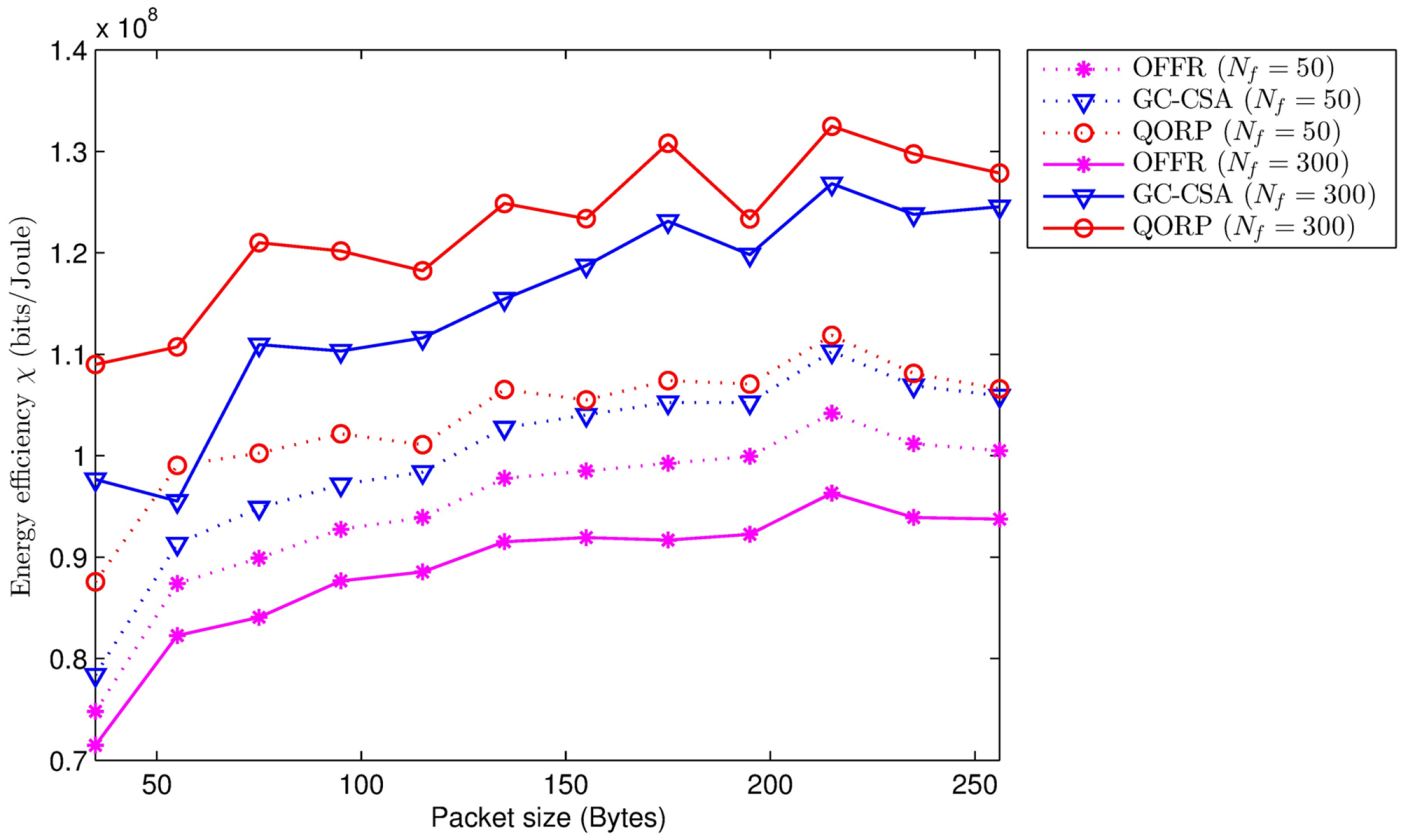
Energy efficiency vs. packet size.

When the packet size increases, the reduction part 10log_10_(*N*) + Δ_*MCS*_ decreases. In addition, the energy efficiency *χ* increases due to high modulation and encoding performance (see Fig 6). When the number of FeNBs increases from 50 to 300, the inter-cell interference in the OFFR algorithm significantly increases, leading to a reduction in energy efficiency. However, the GC-CSA algorithm and our proposed algorithm mitigate most of the interference, thus providing better performance. The results show that our QORP algorithm has the best energy efficiency, *χ*_*QORP*_ = 1.13 × 10^8^ bits/Joule (i.e., the average value), compared to *χ*_*GC* − *CSA*_ = 1.07 × 10^8^ bits/Joule and *χ*_*OFFR*_ = 0.92 × 10^8^ bits/Joule. The improvement achieved through use of the QORP algorithm compared to the GC-GSA and OFFR algorithms is 5.27% and 23.09%, respectively.

**Fig 7:**
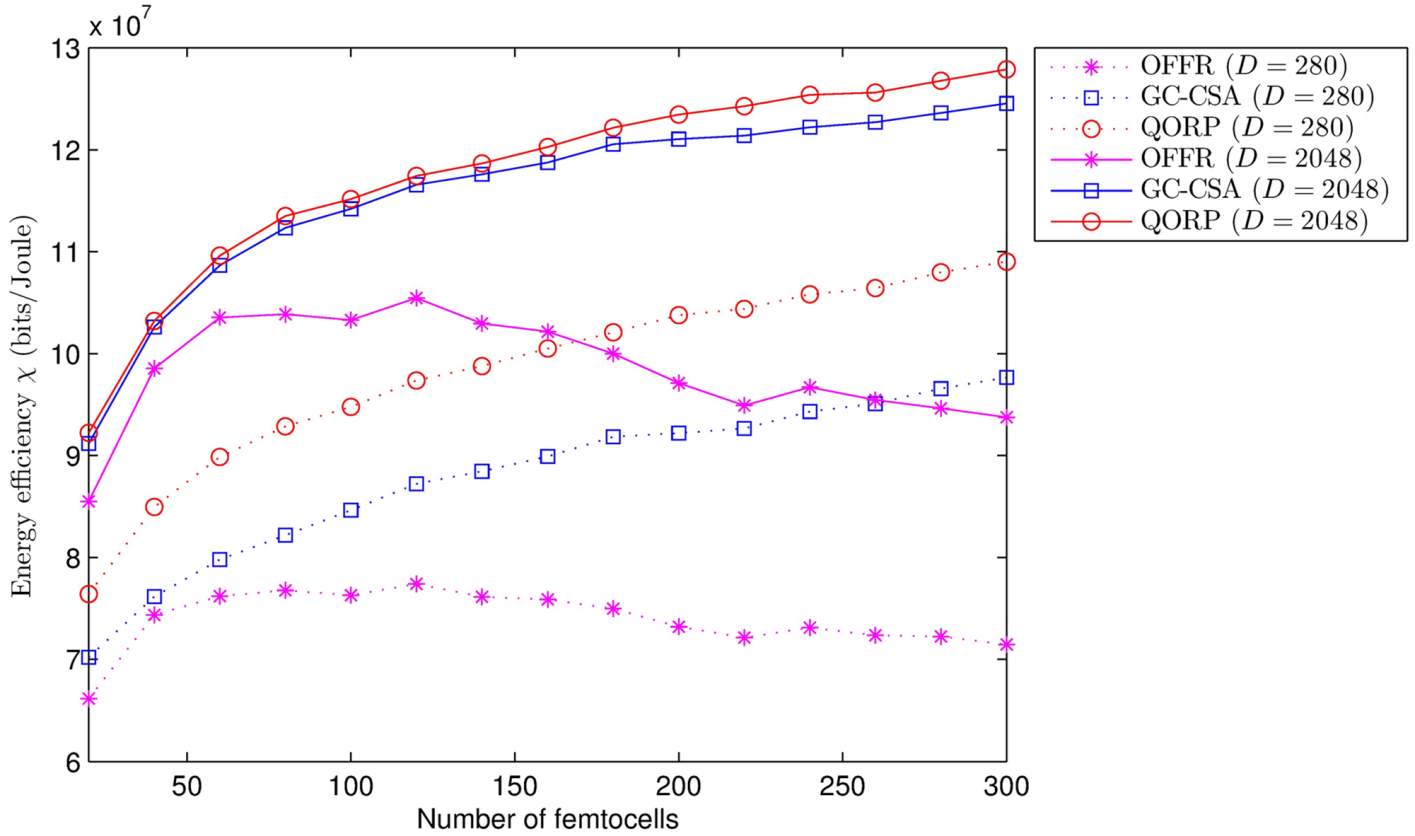
Energy efficiency vs. number of FeNBs.

Fig 7 shows the relationship between the energy efficiency and the number of FeNBs (i.e., it represents the different environment conditions such as the different numbers and positions of the FeNBs, which result in different values for the receiver power and SINR at the sensing nodes). First, the performance of OFFR increases when the number of FeNBs increases due to the low inter-cell interference, and it achieves its best performance when the number of FeNBs is around 120. If the number of FeNBs is more than 120, the performance decreases because the high inter-cell interference negatively impacts the channel quality. The GC-CSA and QORP algorithms provide better energy efficiency when the number of FeNBs is high because they mitigate most of the interference components.

**Fig 8:**
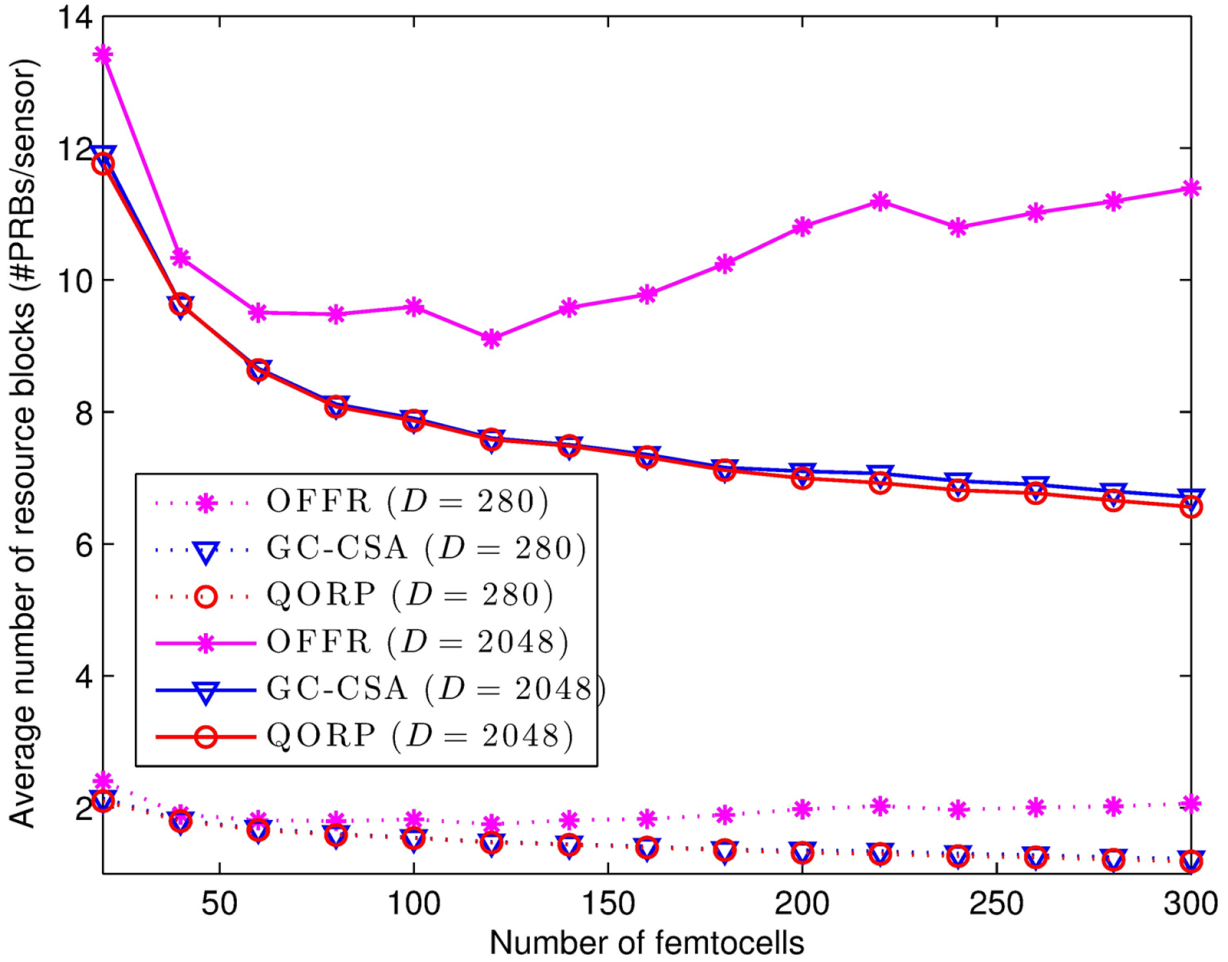
Resource block allocation depends on the number of FeNBs.

As presented in Eq 11, the criteria to determine the interference among FeNBs adapt with the optimal channel quality required by the existing sensing nodes in the network. Therefore, the QORP algorithm provides better energy efficiency for the sensing nodes without any increment of the number of assigned resource blocks for data transmission. Fig 8 shows that the number of assigned resource blocks decreases when the number of FeNBs increases. The QORP reduces the number of assigned resource blocks by 25.98% in comparison with the OFFR algorithm. This result is even better than the performance of GC-CSA because the QORP also reduces the interference with the uplink when the sensing nodes reduce their transmit power. The numerical simulation results are summarized in Table 4.

**Table 4:** The average number of assigned resource blocks for each sensing node.

Packet size (Bytes)	OFFR (PRBs/sensing node)	GC-CSA (PRBs/sensing node)	QORP (PRBs/sensing node)
35	1.937	1.481	1.457
256	10.496	7.823	7.745

**Fig 9:**
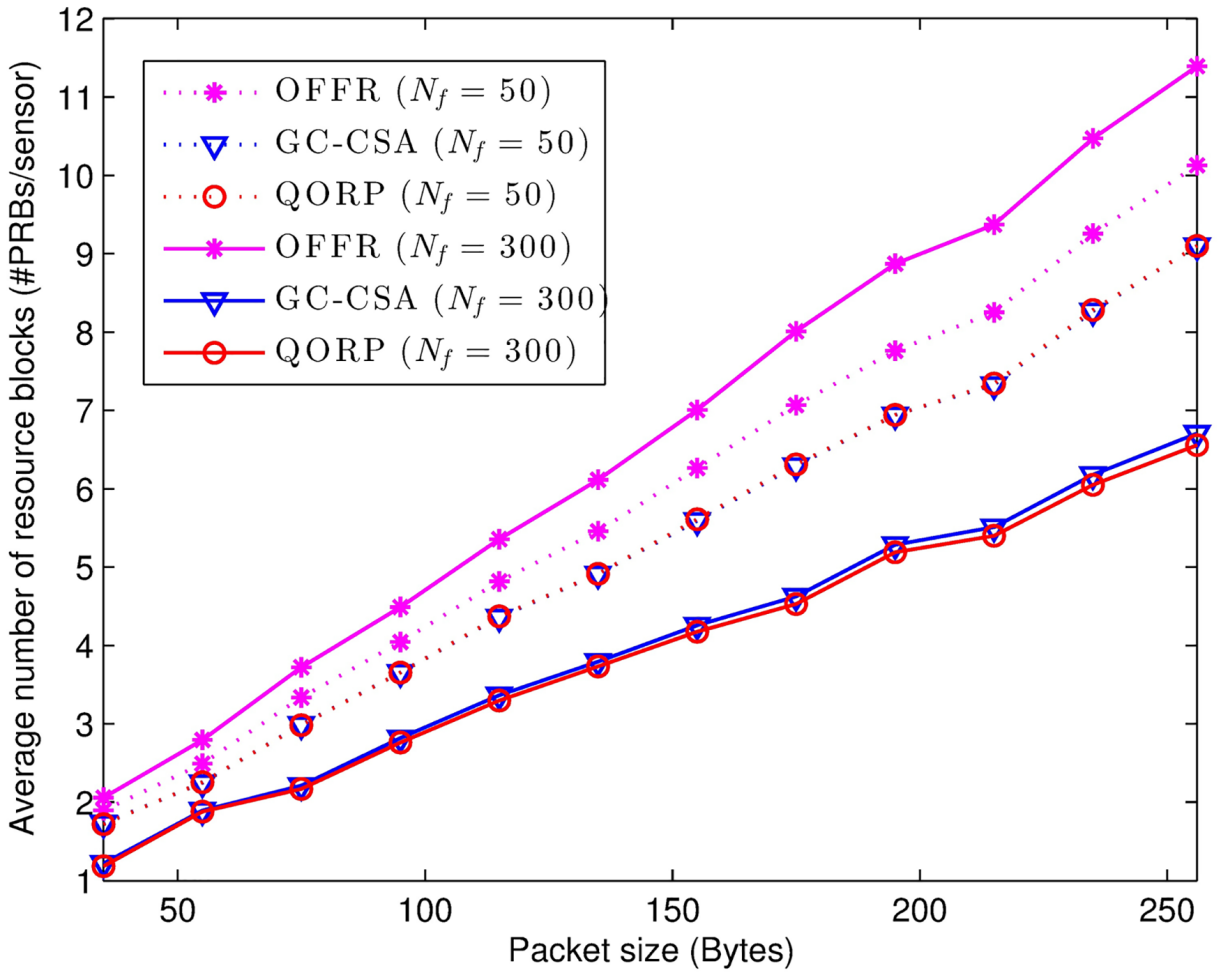
Resource block allocation depends on the packet size.

Fig 9 presents the relationship between the average number of assigned resource blocks and the packet size. The results show the effectiveness of the QORP algorithm in comparison with the OFFR algorithm, which increases from 9.69% to 42.45% when the packet size increases from 35 Bytes to 256 Bytes. The QORP algorithm achieves better performance with large packet sizes in terms of the assignment of the required resource blocks for each sensing node.

## 6 Concluding remarks and future work

Although the LTE-M infrastructure introduces many benefits for the proliferation of IoT sensing platforms, the challenges are also considerable, especially in terms of the energy efficiency. In this paper, we proposed adaptive MCS selection and resource planing algorithms to achieve the minimum transmit power in IoT sensing nodes. The proposed adaptive MCS-PHR selection scheme determines the optimal MCS and #PRBs, at which the sensing data is sufficiently packetized into the transport block size. Based on the optimal MCS, the quantity-oriented resource planning scheme adaptively reassigns appropriate resources between the FeNBs. Intensive analysis and numerical evaluation show that our proposed algorithms achieve better energy efficiency compared to existing algorithms, especially when the transmission packet size is small, which is popular in IoTSPs. The transmission energy and the number of assigned PRBs decreased by up to 23.09% and 25.98%, respectively. In spite of achieving considerable improvements, the proposed algorithm maintains its complexity of $O(1.1571^{n})$ [28] compared to the GC-CSA ($O(1.9464^{n})$ [33]) and the OFFR (O(1) [34]). As a future research direction, we plan to extend this research to consider multiple types of IoT devices with different data transmission requirements.

## Supporting information

S1 Dataset Input parameters of the network model for the simulation. (ZIP)
